# Prevalence and Predictive Clinical Characteristics of Metabolically Healthy Obesity in Obese Children and Adolescents

**DOI:** 10.7759/cureus.35935

**Published:** 2023-03-09

**Authors:** Ismail Dundar, Aysehan Akinci

**Affiliations:** 1 Pediatric Endocrinology Department, Inonu University, Faculty of Medicine, Malatya, TUR

**Keywords:** obesity, childhood obesity, metabolically unhealthy obesity, metabolically healthy obesity, metabolic syndrome

## Abstract

Background: The increasing prevalence of childhood obesity and accompanying comorbidities all over the world constitutes one of the most important public health problems of the changing world. The frequency and causes of the metabolically healthy obesity (MHO) phenotype in children is not clear.

Objective: The objective is to determine the prevalence of the MHO phenotype in obese Turkish children and adolescents and to identify clinical and biochemical indicators for this phenotype.

Methods: Eight hundred forty-seven obese children and adolescents, aged 3-18 years with BMI-SDS >+2 SD from the obesity outpatient clinic were included. Demographic, anthropometric, and physical examination information was collected from patient medical files. In addition, obesity-related comorbidities and results of laboratory tests were obtained. For study purposes, obese patients with no cardiometabolic risk factors were accepted as MHO, and those with ≥1 cardiometabolic risk factor were considered metabolically unhealthy obese (MUO). MHO was defined according to Damanhoury's criteria.

Results: Out of 847 children (mean age 10.6±3.4 years) who met the study criteria, 289 (34.1%) were diagnosed with MHO. Being younger, prepubertal, having relatively low BMI, low waist/hip ratio, low insulin resistance (HOMA-IR) index, high high-density lipoprotein, low triglyceride, low fasting insulin and glucose levels, low uric acid and low alanine transaminase (ALT) levels were associated with MHO.

Conclusions: The MHO phenotype was present in just over a third of this obese pediatric cohort. The most important factors associated with MHO; age, waist-hip ratio, and BMI were determined.

## Introduction

With the increasing global childhood obesity, the prevalence of hormonal, metabolic, and cardiovascular comorbidities in children and adolescents is also increasing. This situation has become one of the important public health problems of the changing world [1,2]. The increasing prevalence of metabolic syndrome (MetS), which develops due to increasing childhood obesity, leads to a possible decrease in life expectancy. Increasing MetS and obesity-related comorbidities mean that today's children and adolescents may have a shorter life expectancy than their parents [1-3]. Childhood obesity causes comorbidities such as high blood pressure, dyslipidemia, impaired glucose tolerance, type 2 diabetes, obstructive sleep apnea, non-alcoholic fatty liver disease (NAFLD), and MetS at any time of life [4,5].

The concept of metabolically healthy obesity (MHO) has been proposed for both adults and children [6,7]. Although these children are obese, they have normal insulin sensitivity, normal blood pressure and preserved glucose regulation, normal serum lipid level, and normal liver function. At the same time, the endocrine, immune, and inflammatory profiles of these obese children are intact [7-11]. While the prevalence of MHO reportedly varies between 10% and 30% in adult obese individuals, this is reported to be between 20.9% and 49.3% in children and adolescents [6,9,11,12].

Mechanisms associated with preserved insulin sensitivity, low visceral adipose tissue, low adipose tissue infiltration, normal adipose tissue, and a physiological adipokine secretion model, as well as good physical activity, have been suggested in the pathogenesis of MHO [10]. However; regarding the pathophysiology of MHO; uncertainties remain about predicted environmental factors and genetic determinants. The applicability of the concept of MHO in children still continues to be discussed [7,10,12,13]. Despite differences in diagnostic criteria for MHO in children, all studies have reported that a percentage of obese children demonstrate a "positive" metabolic state.

In 2018, MHO was defined according to the Damanhoury criteria [8]. Using WHO growth charts, Obesity is defined as a BMI z-score greater than +2 standard deviation score (SDS) [8,14]. In our study, obese patients who met Damanhoury criteria were accepted as MHO. However, in the Damanhoury et al consensus, there is no agreement on whether the threshold of fasting plasma glucose is ≤100 mg/dL as a criterion for MHO euglycemia in children [8].

There are few studies about obese children's MHO in Turkey [15]. This study was conducted to determine the frequency of MHO in obese Turkish children and adolescents. Another aim of this study is to determine potential clinical and biochemical indicators for MHO and metabolically unhealthy obesity (MUO) phenotypes.

## Materials and methods

Study setting and participants

Our study was designed as a single-center, prospective study. Children diagnosed with obesity in the pediatric endocrinology clinic between March 2013 and September 2018 were selected for the study. Data of all patients were extracted from computerized databases and archive files. Inclusion criteria were: (1) age, 3-18; (2) BMI-SDS > +2 SD [14]; and (3) absence of chronic disease. Exclusion criteria from the study: (1) the presence of type 1 diabetes, short stature, and/or hypothyroidism (Cases with subclinical hypothyroidism were included in the study), Cushing's disease, or patients using topical or systemic steroid therapy; (2) monogenic or syndromic obesity; and (3) missing data on MHO parameters in the records.

Clinical and biochemical variables

Anthropometric measurements and physical examination of the cases were performed. The puberty status was made in accordance with the Tanner criteria. The armpit and neck were screened for acanthosis nigricans. Skin findings (striae) in the abdomen and hips were evaluated. Height was measured using the Harpenden Stadiometer (with 0.1 cm measurement precision) (Holtain Ltd, Crymych, UK). Weight measurement was made with a sensitive scale up to 0.1 kg in the morning, hungry, without shoes, and with underwear (BMI SECA). BMI was measured as (kg [weight]/m^2^ [height]). Waist circumference was measured at the level of the umbilicus with a flexible measuring tape. Hip circumference was measured at the iliac crest level with a flexible tape measure. Blood pressure was measured on the right arm with a standard mercury sphygmomanometer after resting for 5-10 minutes in a quiet environment. Pubertal status was examined by Tanner staging. Testicular volume of ≥4 mL in males and ≥2 breast development (Tanner stage 2) in females according to Tanner's stage were accepted as markers indicating the onset of puberty [16,17].

Serum blood glucose was measured by the glucose oxidase method and lipids were measured with an Olympus AU 2700 device (Beckman Coulter, USA). Insulin level was determined by chemiluminescence method in Roche Modular Analytics E-170 immunoassay analyzer (Roche Diagnostics, Indianapolis, IN, USA). Serum thyroid stimulating hormone (TSH), free triiodothyronine (FT3) and free thyroxine (FT4) levels were analyzed by chemiluminescence method on an Elecsys 2010 modular analytical E170 immunoassay autoanalyzer (Roche Diagnostics, Indianapolis, IN, USA). For insulin resistance: “Homeostasis model assessment” (HOMA) formula (fasting insulin [mIU/mL] x fasting glucose [mmol/L]/22.5) was used [18].

Data collection and definitions

Information on age, sex, puberty, gestational age and birth weight, presence of gestational diabetes mellitus, duration of breastfeeding, anthropometric measurements (weight, height/waist/hip ratio), acanthosis nigricans, presence of striae, and history of snoring were obtained from medical records. Hypertension, abnormal glucose metabolism (IFG and IGT), NAFLD, subclinical hypothyroidism and results of laboratory tests (fasting lipid profile, fasting glucose and insulin levels, HbA1c, liver function tests) and uric acid (UA) levels were also obtained from medical records. Blood pressure was evaluated according to age and gender using Centers for Disease Control and Prevention (CDC) data [19].

BMI-SD values were calculated according to age and gender using World Health Organization (WHO) data [14]. MHO was diagnosed according to Damanhoury's criteria. Obese patients who met all of these criteria were accepted as MHO [8]. The MHO criteria are shown in Table 1. Cardiometabolic risk factors by age and sex: (1) blood pressure >90th percentile [20]; (2) serum triglyceride level >150 mg/dL or (3) high-density serum lipoprotein (HDL) cholesterol <40 mg/dL [6]; and (4) fasting serum blood glucose >100 mg/dL [21]. Patients with cardiometabolic risk factor clustering were defined as having MUO. MUO was divided into two subgroups, MUO-1 for patients with one cardiometabolic risk factor and MUO-2 for patients with ≥2 cardiometabolic risk factors.

**Table 1:** Consensus-based definition of MHO in children (8).

Children who met all the following criteria were classified as MHO, provided BMI-SDS>+2 SD (using the WHO growth charts)	
1. HDL	>40 mg/dL (>1.03 mmol/L)
2. Triglycerides	≤150 mg/dL (≤1.7 mmol/L)
3. Blood pressure (systolic and diastolic)	≤90^th^ percentile
4. A measure of glycemia	Fasting plasma glucose ≤100 mg/dL (≤5.6 mmol/L)

A HOMA-IR value above 2.5 in prepubertal children and above 3.16 in the pubertal group was considered as insulin resistance [18,22]. The upper abdominal ultrasonographic evaluation was performed to assess hepatosteatosis using an ultrasound device (GE LOGIC S8, USA). Hepatosteatosis was divided into 3 stages by liver ultrasonography. Stage 1 (mild), stage 2 (moderate) and stage 3 (severe) hepatosteatosis [23]. In our center's laboratories, the limit values for TSH are 0.35-5.5 mIU/mL, the limit values for fT4 are 0.61-1.32 ng/dL, and the limit values for fT3 are between 1.9-4.2 pg/mL [24].

Ethics

This study was approved by the Ethics Committee of Inonu University Faculty of Medicine (Approval number: 2021/2654). The study was conducted in accordance with the Declaration of Helsinki.

Statistical analysis

The Statistical Package for the Social Sciences 17.0 (SPSS, Inc., Chicago, IL, USA) was used for statistical analysis (SPSS, Inc., Chicago, IL, USA). Values were summarized as mean ± standard deviation and minimum-maximum. The chi-square test was used for categorical variables. The normal distribution of the data was checked by Kolmogorov-Smirnov test. Student t-test and Mann-Whitney U test were used to compare the parametric values of MHO and MUO groups. Significance is stated as P ≤ 0.05.

## Results

Eight hundred forty seven patients (496 males - 58. 5%) aged 3-18 years were analyzed. The BMI-SDS range was 2.01-5.91 and the median value was 2.3. Of the patients, 406 (47.9%) were Tanner stage 1, and 441 (52.1%) were Tanner stage ≥2. The mean ± SD age was 10.6±3.4 years, being 10.5±3.6 years in girls and 10.7±3.3 years in boys.

MHO was detected in 34.1% of the cases. While this rate was 33.7% in boys, it was 34.8% in girls, which was not significantly different. The MHO prevalence was 44.6% and 24.5% in prepubertal and pubertal children, respectively (p=0.001). MUO was 55.4% in prepubertal cases and 75.5% in pubertal cases (p=0.001). While insulin resistance was 55.7% in all cases, it was 61% in pubertal cases and 50% in prepubertal cases (p=0.001). Anthropometric, biochemical, and comorbidity data of the study group according to sex and puberty status are presented in Table 2.

**Table 2:** Anthropometric, laboratory and obesity-related comorbidity data of our study cohort according to gender and pubertal status *comparison by gender, ** comparison by puberty. Abbreviations: BMI-SDS: body mass index-standard deviation score, LGA: large-for-gestational-age, AGA:  appropriate-for-gestational-age, SGA: small-for-gestational age, GDM: gestational diabetes mellitus, IFG:  impaired fasting glucose, NAFLD: non-alcoholic fatty liver disease, MHO: metabolically-healthy obesity, LDL: low density lipoprotein, HDL: high density lipoprotein, HbA1c: glycated hemoglobin, HOMA-IR: homeostasis model assessment insulin resistant, ALT: alanine transaminase, AST: aspartate aminotransferase.

	All (n= 847)		Males (n= 496)	Females (n = 351)	p*	Prepubertal (n = 406)	Pubertal (n = 441)	p**
Age (yr)	10.6±3.4		10.7±3.3	10.5±3.6	NS	7.9±2.1	13.1±2.3	<0.001
BMI	28.6±4.6		28.5±4.3	28.8±5.0	NS	25.7±3.3	31.3±4.0	<0.001
BMI SDS	2.4±0.4		2.4±0.5	2.3±0.3	0.006	2.4±0.4	2.2±0.3	<0.001
WHR	0.95±0.03		0.94±0.03	0.96±0.03	<0.001	0.96±0.03	0.95±0.03	<0.001
AGA,/LGA,/SGA, %	75.4/15.8/8.8		72.0/19.6/8.4	80.2/10.5/9.3	0.02	76.5/14.8/8.7	74.4/16.7/8.9	NS
Birth weight (kg)	3.4±0.77		3.3±0.7	3.5±0.8	0.002	3.4±0.7	3.4±0.8	NS
Breastfeeding time (month)	13.6±10		14.3±10.2	12.6±9.6	0.023	13.8±10.1	13.5±10.0	NS
GDM,%	9.7		10.1	9.1	NS	11.3	80.2	NS
Acanthosis nigricans, %	55.8		55.0	57.0	NS	37.2	68.1	<0.001
Striae, %	49.8		46.6	54.4	0.025	25.6	74.4	<0.001
Snoring, %	30.4		34.7	24.0	0.002	29.4	31.3	NS
Presence of obesity-related comorbidities								
Insulin resistance, %		55.7	58.3	52.1	0.045	50.0	61.0	0.001
Dyslipidemia, %		56.7	58.6	54.5	NS	49.3	63.7	<0.001
Hypertension, %		20.8	19.6	22.5	NS	16.0	25.2	0.001
IFG, %		7.2	9.1	4.6	0.012	8.1	6.1	NS
NAFLD (Stage 1-3), %		41.9	42.9	40.5	NS	34.8	48.6	0.001
Subclinical hypothyroidism, %		7.1	6.3	8.3	NS	7.4	7.0	NS
MHO		34.1	33.7	34.8	NS	44.6	24.5	0.001
Patient-related biochemical data								
Total cholesterol (mg/dL)	167.4±33.7		168.5±35.5	164.4±30.8	NS	167.0±32.9	167.7±34.5	NS
LDL-cholesterol (mg/dL)	100.3±29.4		100.7±30.9	99.3±27.1	NS	100.6±28.2	100.1±30.5	NS
HDL-cholesterol (mg/dL)	44.3±12.4		43.9±11.7	44.4±13.2	NS	46.0±12.9	42.5±11.6	<0.001
Triglycerides (mg/dL)	123.4±58.4		123.7±60.2	123.2±55.8	NS	113.0±53.8	132.8±60.8	<0.001
Fasting glucose (mg/dL)	89.2±8.1		90.0±8.3	88.0±7.6	<0.001	89.0±8.3	89.3±7.9	NS
Fasting insulin (mcU/mL)	18.4±10.2		17.8±9.6	19.1±11.0	NS	14.1±8.6	22.1±10.1	<0.001
HbA1c	5.5±0.4		5.4±0.5	5.4±0.4	NS	5.4±0.4	5.5±0.4	0.001
HOMA-IR	4.0±2.4		3.9±2.2	4.1±2.5	NS	3.1±2.0	4.8±2.3	<0.001
Uric acid (mg/L)	5.1±1.3		5.2±1.4	4.9±1.1	<0.001	4.5±1.0	5.5±1.3	<0.001
AST (U/L)	24.3±10.0		25.8±11.5	22.5±7.1	<0.001	26.4±11.4	22.5±8.1	<0.001
ALT (U/L)	25.1±15.6		27.8±18.8	21.2±9.4	<0.001	24.7±14.7	25.1±16.5	NS

**Figure 1:**
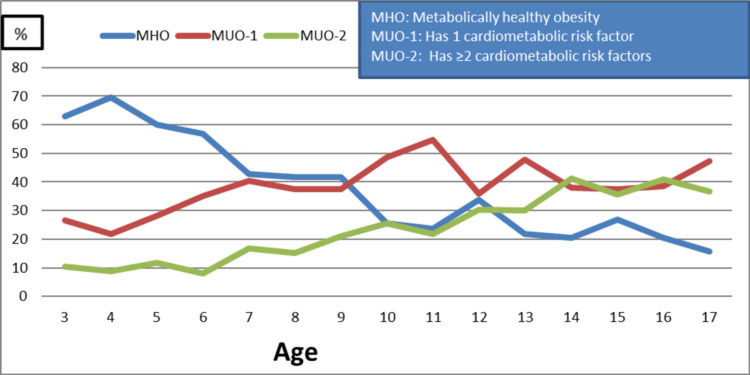
Percentage change of MHO, MUO-1, and MUO-2 according to patient age

The MHO group consisted of 289 patients, of whom 167 (57.8%) were male (34.1% of the entire cohort). The MUO-1 group consisted of 346 patients (40.8% of the whole cohort), of whom 207 (59.8%) were male. The MOU-2 group consisted of 212 subjects (25.1% of the entire cohort), of which 122 (57.5%) were male. The percentage change of MHO, MUO-1, and MUO-2 according to the age of the patients is given in Figure 1. Table 3 shows comparative anthropometric and laboratory values of MHO and MUO cases.

Regarding MHO, being younger, prepubertal, having normal fasting insulin and glucose levels, low BMI, low waist/hip ratio, low triglyceride, high HDL-C, and low HOMA-IR were associated with low UA and low ALT levels (Table 3).

**Table 3:** Anthropometric, laboratory and obesity-related comorbidity data of our study cohort according to MHO and MUO status at diagnosis p*; Comparison of MHO with the group with one cardiometabolic risk factor, p**; Comparison of MHO and group with multiple cardiometabolic risk factors. Abbreviations: MHO: metabolically-healthy obesity, MUO: metabolically unhealthy obesity, LGA: large-for-gestational-age, AGA: appropriate-for-gestational-age, SGA:  small-for-gestational age, BMI-SDS: body mass index-standard deviation score, GDM: Destational diabetes mellitus, IFG: impaired fasting glucose, NAFLD: Non-alcoholic fatty liver disease, NAFLD: Non-alcoholic fatty liver disease, LDL: low density lipoprotein, LDL: low density lipoprotein, HbA1c: glycated hemoglobin, HOMA-IR: homeostasis model assessment insulin resistant, ALT: alanine transaminase, AST: aspartate aminotransferase.

	MHO (n = 289) (No cardiometabolic risk factors)	MUO-1 (Presence of one cardiometabolic risk factor) (n=346)	MUO-2 (Presence of multiple cardiometabolic risk factors (n=212)	p*	p**
Age (yr)	9.5±3.4	10.8±3,2	11.7±3.4	<0.001	<0.001
Gender Males, n (%) Females, n (%)	167 (57.8) 122 (42.2)	207 (59.8) 139(40.2)	122 (57.5) 90 (42.5)	NS	NS
Puberty				<0.001	<0.001
Prepubertal, n (%)	181(62.6)	158 (45.7)	67(31.6)		
Pubertal, n (%)	108 (37.4)	188 (54.3)	145 (68.4)		
AGA,/LGA,/SGA, %	76.8/14.1/9.1	74.2/16.1/9.7		NS	NS
BMI	26.9±4.1	28.7±4.4	30.7±4.8	<0.001	<0.001
BMI SDS	2.3±0.4	2.3±0.3	2.39±0.4	NS	NS
Waist Hip Ratio	0.95±0.02	0.96±0.03	0.97±0.03	0.021	<0.001
Breastfeeding time (month)	13.7±9.2	13.2±10.4	14.2±10.5	NS	NS
GDM, %	10.4	9.8	8.5	NS	NS
Acanthosis nigricans , %	46.4	56.4	67.9	0.012	<0.001
Striae, %	40.5	50.9	60.8	0.009	<0.001
Snoring, %	26.7	30.8	36.4	NS	0.042
Insulin resistance, %	18.7	65.3	69.3	<0.001	<0.001
Dyslipidemia, %	18.0	65.3	95.8	<0.001	<0.001
Hypertension, %	0	25.4	30.0	<0.001	<0.001
IFG, %	0	8.7	13.2	<0.001	<0.001
NAFLD (Stage 1-3), %	37.8	43.7	45.5	NS	0.039
Subclinical hypothyroidism, %	5.5	7.5	8.5	NS	NS
Total cholesterol (mg/dL)	168.2	165.6	169.3	NS	NS
LDL-cholesterol (mg/dL)	101.2	101.1	97.8	NS	NS
HDL-cholesterol (mg/dL)	51.3	43.0	36.7	<0.001	<0.001
Triglycerides (mg/dL)	91.6	116.8	177.0	<0.001	<0.001
Fasting glucose (mg/dL)	87.7	89.6	90.4	0.002	<0.001
Fasting insulin (mcU/mL)	15.2	18.5	22.1	<0.001	<0.001
HbA1c	5.4	5.4	5.4	NS	NS
HOMA-IR	3.2	4.1	4.9	<0.001	<0.001
Uric acid (mg/L)	4.7	5.1	5.4	0.001	<0.001
AST (U/L)	24.3	23.4	25.4	NS	NS
ALT (U/L)	24.1	23.1	28.7	NS	0.003

## Discussion

We found the prevalence of MHO to be 34.1% in our cohort of obese patients. While MHO was in the range of 19-21.7% in previous pediatric studies [6,11,25,26], it was reported to be significantly higher at 49.3% in the German pediatric population by Reinehr et al.12 In Turkey, Elmaogullari et al. [15] reported an MHO prevalence of 40.8% and female predominance in obese children. However, in the study of Rocha et al., there was male predominance [27]. There was no sex difference in our study. These differences in MHO prevalence are reported to be due to sex, ethnicity, socioeconomic status, behavioral differences, environmental differences, and the interaction between these variables. Until 2018, the criteria used for the definition of MHO in pediatrics were very diverse and there was no consensus. Differences in diagnostic criteria may also have affected the difference in prevalences. In our study, we used the international consensus-based criteria proposed by Damanhoury et al. [8] in 2018 for MHO as diagnostic criteria.

Puberty is an important period for IR development due to increased adipose tissue [28]. Especially in girls, with the increase in sex steroids, there is an increase in both total fat mass and subcutaneous fat distribution. Differences between superficial fat stores occur during adolescence. Puberty is an important indicator of MUO, and the frequency of MUO increases with puberty [12,29]. In our study, while the proportion of MHO was 44.6% in prepubertal cases, it decreased to 24.5% with puberty, and the prevalence of MHO decreased approximately 1.8 times. We also found a trend of the increasing prevalence of cardiometabolic risk factors with the transition to puberty. A one-year longitudinal study of children with obesity in 2017 showed that entering puberty doubled the risk of transitioning from the MHO to the MUO phenotype while transitioning to the mid- to late-puberty stage almost tripled the probability of transitioning from the MHO to the MUO phenotype [12]. Again, this study showed that early pubertal development indicates being metabolically healthy [12]. In our study, the prevalence of MHO at the age of 3-6 was around 60%, while this rate decreased by around 20% at the age of 15-17. While MHO decreased with age and puberty, MUO increased. Sex hormones and a pubertal spurt of Growth hormones add to insulin resistance. The influence of sex hormones and physiological changes in body composition during puberty development may explain the relationship between the pubertal stage and cardiometabolic changes.

In contrast to MHO, children with MUO had higher BMI SDS, were more overweight, had higher waist circumference (WC), higher insulin resistance index (HOMA-IR), lower insulin sensitivity, and higher pro-inflammatory markers. Regarding the liver profile in this study, ALT and AST serum concentrations were not significantly different between MHO and MUO children [27]. In our study, children with MHO were younger (prepubertal) and had lower BMI, lower waist/hip ratio, lower serum UA levels, and normal insulin levels. Our study found lower ALT levels and less fatty liver in the MHO group. Studies show that high serum UA levels in obese children and adolescents are an important indicator of metabolic unhealthiness [27,30]. In our study, serum UA levels were significantly higher in children with MUO. With this result, high UA may be used as an indicator of poor metabolic control.

Insulin resistance is a very important factor in the development of obesity-related comorbidities and is a complex metabolic process that is still not fully understood to date. Lower insulin resistance, a lower degree of hepatosteatosis, and absence of acanthosis nigricans have been shown to be important determinants of the MHO phenotype [19,27,31]. These findings can be used as predictors for MHO. Margolis-Gil et al. [11] suggested that male sex, high BMI, high HOMA-R, and the presence of acanthosis nigricans are good predictors of MUO development. In our study, low BMI, low insulin resistance, fewer striae, less snoring, and fewer acanthosis nigricans were important indicators for MHO. This indicates a low β-cell reserve. Increased HOMA-IR value may indicate early changes in glucose metabolism in obese children that may be associated with decreased insulin sensitivity.

It is known that a significant proportion of obese individuals do not develop MUO. This presents significant diagnostic challenges associated with the use of obesity as a risk factor for cardiometabolic disease. According to one hypothesis, it is not the total amount of body fat that adversely affects health in obesity, but the distribution of lipids in the body. According to the "adipose expandability hypothesis", when the fat storage capacity of adipose tissue is exceeded, lipid accumulation in non-fat organs will increase, which will lead to IR and apoptosis as a result of lipotoxicity [32]. Studies in obese adolescents have shown that IR is associated with a specific accumulation of abdominal and ectopic fat, independent of total body fat [33]. Weis et al. [34] reported that even if BMI remained constant (due to increased height), increasing amounts of adipose tissue were associated with a decrease in insulin sensitivity and progression from normal glucose response to dysglycaemia on OGTT. Pacifico et al. suggested that early recognition of the metabolic syndrome and implementation of effective treatment are the first important steps toward reducing morbidity and mortality associated with cardiometabolic diseases [35]. Early identification of predictors for the development of MUO and identification and monitoring of patients who may benefit from early intervention will become very important. This is because current screening guidelines do not differentiate between levels of obesity.

The study has some significant limitations. (1) The major limitation of the study is that it is a cross-sectional study evaluating MHO in pediatric obese patients. (2) Lack of data on diet, physical activity, age of onset of obesity, and socio-economic status. (3) The fact that it is a tertiary university hospital may have overestimated the frequency of MUO. Long-term longitudinal studies evaluating MHO in obese children are needed.

## Conclusions

In conclusion, the MHO phenotype was present in 34.1% of obese Turkish children, indicating that screening programs should be implemented more broadly and effectively. Younger age (prepubertal period), lower BMI, low waist/hip ratio, low HOMA-IR, high HDL, low triglyceride, low ALT level, and low UA level are important indicators for MHO. Acanthosis nigricans, striae, snoring, and insulin resistance increase in prevalence with the transition from MHO to MUO. Detection, early intervention, and monitoring of MUO patients who do not have these indicators may help to reduce adult cardiovascular disease risk.
